# Protocol for isolating and culturing neonatal murine cardiomyocytes

**DOI:** 10.1016/j.xpro.2024.103461

**Published:** 2024-11-22

**Authors:** Chiara Bongiovanni, Carmen Miano, Francesca Sacchi, Silvia Da Pra, Irene Del Bono, Stefano Boriati, Gabriele D’Uva

**Affiliations:** 1 Department of Medical and Surgical Sciences, University of Bologna, Via Massarenti 9, 40138 Bologna, Italy; 2 Centre for Applied Biomedical Research (CRBA), University of Bologna, Via Massarenti 9, 40138 Bologna, Italy; 3 IRCCS Azienda Ospedaliero-Universitaria di Bologna, Via Massarenti 9, 40138 Bologna, Italy

**Keywords:** Cell Biology, Cell culture, Cell isolation, Developmental biology

## Abstract

The isolation and culture of neonatal murine cardiac cells are valuable techniques for studying their properties and molecular mechanisms in response to various treatments or conditions. Here, we present a protocol for isolating a high yield of viable neonatal murine cardiac cells, including functional, beating cardiomyocytes. We describe the steps of heart extraction, washing and pre-digestion, digestion, and cell seeding. We detail procedures for mechanical and enzymatic digestions, conducted in a controlled environment within a cell culture incubator.

For complete details on the use and execution of this protocol, please refer to Bongiovanni et al.^1^

## Highlights

• Instructions for isolating neonatal murine cardiac cells in a cell culture incubator
• Steps for easy and efficient enzymatic and mechanical digestion of cardiac tissue
• Guidelines for maximizing viable cardiac cell yield by stable incubator temperature
• Guidelines for minimizing contamination risks through a sterile incubator environment

## Abstract (editor highlights)

Publisher’s note: Undertaking any experimental protocol requires adherence to local institutional guidelines for laboratory safety and ethics.

## Graphical abstract

**Figure:**
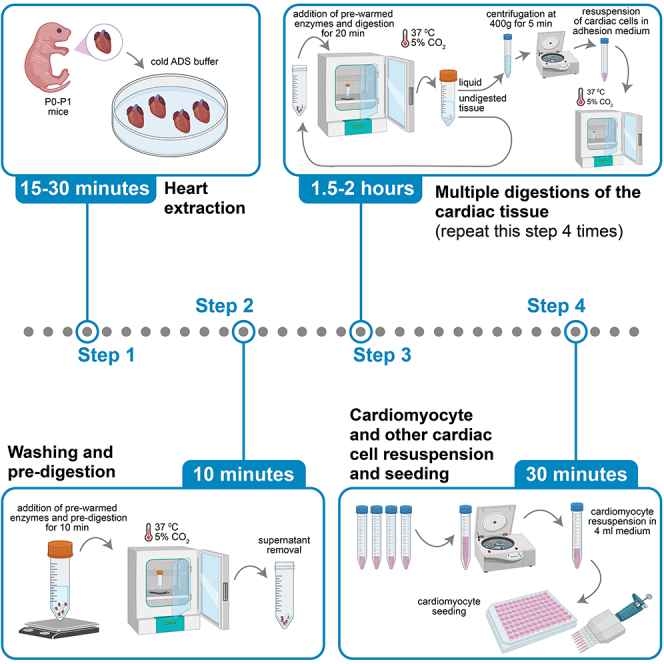

## Before you begin

Reliable and reproducible cardiac cell culture methods are essential for advancing research in cardiac development, pathology and regeneration. While numerous protocols exist for isolating viable mammalian neonatal cardiac cells through enzymatic and mechanical digestions,^2^^,^^3^^,^^4^^,^^5^^,^^6^^,^^7^ many present significant challenges that our approach aims to overcome.

For instance, some protocols involve placing the tissue, digesting enzymes, and a magnetic stir bar into reusable glass tubes or Erlenmeyer flasks, which are then immersed in a water-filled glass vessel on a heated magnetic stirrer to maintain the water temperature at 37°C while stirring.^7^ However, maintaining the water temperature at a stable temperature can be challenging, requiring constant manual monitoring with a thermometer. Additionally, the submerged glass tubes or flasks are often unstable, increasing the risk of contamination of the tissue by the surrounding water. To circumvent these issues, we previously attempted placing the tube containing the tissue, enzymes, and magnetic stir bar directly on a heated magnetic stirrer set to 37°C or slightly higher. Although this strategy provided greater stability and reduced the risk of water contamination, it caused a decrease in cell yield due to suboptimal temperature distribution, with excessive heat accumulating at the bottom of the tube or flask and cooler temperatures in the upper part of the liquid.

Other protocols rely on enzymatic digestion in a heated shaker,^3^^,^^5^^,^^6^ eliminating the need for a magnetic stirrer and bar. However, achieving proper mechanical agitation of the tissue using this method can be challenging, which may also compromise cell yield.

Some protocols emphasize mechanical tissue dissociation, either by cutting the heart tissue directly or using specialized equipment.^2^ However, vigorous mechanical dissociation can lead to increased physical damage to cardiomyocytes, resulting in a lower overall yield, often less than 500,000 cells per mouse heart.^2^

Finally, certain protocols involve the use of sophisticated and costly equipment, such as the Langendorff apparatus.^4^

Here, we describe a simple and reliable protocol for isolating and culturing a high yield of primary cardiac cells, including viable cardiomyocytes, from mouse models at the neonatal stage (postnatal day 0 or 1). A key advantage of our protocol is that the mechanical and enzymatic digestion is carried out within a CO_2_ cell culture incubator, the same controlled environment in which the isolated cardiac cells will later be cultured. This setup maintains a constant temperature of 37°C, optimizing the activity of the digesting enzymes and reducing digestion time while minimizing damage and stress to the cardiac cells during isolation. Additionally, the sterile environment of the incubator further lowers the risk of contamination. Moreover, using sterile, single-use, self-standing conical tubes offers several advantages. First, it eliminates the need for autoclaving glass flasks or tubes. Second, it provides a more stable placement on the stirrer, removing the instability and contamination risks associated with water immersion. Finally, the conical tubes allow the cardiac tissue to move more freely, promoting more efficient digestion.

The procedure involves successive enzymatic digestions using Collagenase A and Pancreatin, resuspended in a saline buffer known as ADS, combined with mechanical disaggregation of the cardiac tissue by rotating a magnetic bar through a stirrer housed within a cell culture incubator set with standard parameters (37°C and 5% CO_2_). Once isolated and cultured using this protocol, neonatal cardiomyocytes spread out, contract and continue to express sarcomere proteins, including troponin I and T. Additionally, they maintain a low rate of proliferation, making them a useful *in vitro* model for analyzing cardiomyocyte proliferative and regenerative ability, as previously demonstrated by our research group.^1^^,^^8^

### Institutional permissions

Animal procedures described in this protocol were performed in compliance with the Animal Care and Use Committee of the University of Bologna (Italy).

Researchers using this protocol must obtain ethical permission from an Animal Ethics Committee and their institution.

### Preparation of ADS buffer

**Timing: 2 h**

ADS buffer is a saline solution used for washing extracted hearts and for subsequent enzymatic digestion.

1. Dissolve the following compounds in distilled H_2_O:
  a. 3.095 g of NaCl (106 mM, Sigma 7647-14-5).
  b. 2.385 g of HEPES (20 mM, Sigma 7365-45-9).
  c. 0.0568 g of Na_2_HPO_4_ (0.8 mM, Sigma).
  d. 0.197 g of KCl (5.3 mM, Sigma 7447-40-7).
  e. 0.049 g of MgSO_4_×7H_2_O (0.4 mM, Sigma 7487-88-9).
  f. 0.456 g of Glucose (5 mM, Sigma 50-99-7).
2. Adjust the pH to 7.4 with NaOH and add distilled H_2_O to reach the desired volume.
3. Filter the solution using a 0.22 μm filter in a biosafety cabinet and store at 4°C.

**CRITICAL:** The ADS buffer must be used cold and can be stored for up to 2 weeks.

### Preparation of enzymatic digestion solution (collagenase-pancreatin enzyme solution)

**Timing: 30 min**

Cardiomyocytes and other cardiac cells are isolated from whole cardiac tissue by mechanical and enzymatic digestion. The enzyme solution used in this protocol contains Collagenase A (0.45 mg/mL) to degrade the extracellular matrix, and Pancreatin (1 mg/mL), a mixture of amylase, trypsin and lipase enzymes that facilitate protein hydrolysis.

4. Transfer 22.5 mg Collagenase A (Roche 103586) and 50 mg Pancreatin (Sigma P-1750) to a 50 mL tube kept in ice.
5. Add 50 mL of cold ADS buffer and vortex the mixture vigorously until the components are well mixed, though it may not become completely clear.
6. Filter the solution through a 0.22-μm syringe filter in a biosafety cabinet and keep the solution on ice.

**CRITICAL:** Prepare a fresh solution for each isolation. 50 mL of enzymes are sufficient for digesting up to 8–10 hearts.

### Preparation of cardiac cell adhesion medium

**Timing: 15 min**

The cardiac cell adhesion medium consists of a DMEM-F12 with stable Glutamine and 15 mM HEPES, supplemented with fetal bovine serum (FBS), horse serum (HS), penicillin/streptomycin, non-essential amino-acids, glutamine and sodium pyruvate. This medium is also used to store digested cardiac cells at 37°C during the isolation procedure and for the first 2 days of cardiac cell culture.

7. In a biosafety cabinet, prepare the cardiac cell adhesion medium by adding the following components:
  a. 81% DMEM-F12 with stable Glutamine and 15 mM HEPES.
  b. 10% fetal bovine serum (FBS).
  c. 5% horse serum (HS).
  d. 1% penicillin/streptomycin (100X).
  e. 1% glutamine (200 mM).
  f. 1% non-essential amino-acid solution (100X).
  g. 1% sodium pyruvate (100 mM).
8. Filter the medium through a 0.22 μm syringe filter.

***Note:*** Before starting the isolation procedure, pre-warm the adhesion medium in a water bath at 37°C.

### Preparation of gelatin solution

**Timing: 1 day**

A 0.1% gelatin solution is used as a coating treatment for polystyrene cell culture plates to facilitate murine cardiomyocyte adhesion. The plates should be pre-treated with the gelatin solution for at least 1 h in a 37°C incubator before seeding the cardiomyocytes.

9. Prepare a solution in a glass bottle by dissolving 1 mg of gelatin (Sigma G9391) per mL of distilled water. For example, add 500 mg of gelatin to 500 mL of distilled water in a 500 mL glass bottle. Shake gently to mix.
10. Dissolve and sterilize the solution by autoclaving.
11. Allow the solution to cool to room temperature, then store it at 4°C.

**CRITICAL:** The gelatin solution takes a considerable amount of time to cool. Therefore, it is recommended to prepare the solution at least one day before use. Store the solution for a maximum of one month.

## Key resources table

**Table:** 

REAGENT or RESOURCE	SOURCE	IDENTIFIER
**Antibodies**		

Rabbit polyclonal anti-cardiac Troponin I antibody	Abcam	Cat# ab47003, RRID: AB_869982
Mouse monoclonal anti-BrdU antibody	DSHB	Cat# G3G4, RRID: AB_1157913

**Chemicals, peptides, and recombinant proteins**		

DMEM F12 with stable glutamine and 15 mM HEPES	Aurogene	AU-L0092-500
Fetal bovine serum (FBS)	Gibco - Life Technologies	10270106
Horse serum (HS)	Gibco	16050122
Penicillin/streptomycin (100x)	Euroclone	ECB3001D
Glutamine (200 mM)	Sigma-Aldrich	G7513, CAS:56-85-9
MEM non-essential amino acids solution (100x)	Gibco - Life Technologies	11140050
Sodium pyruvate (100 mM)	Gibco - Life Technologies	11360070
Sodium chloride (NaCl)	HiMedia	GRM031, CAS:7647-14-5
HEPES	HiMedia	MB016, CAS:7365-45-9
Sodium phosphate dibasic (Na_2_HPO_4_)	HiMedia	MB024, CAS:7558-79-4
Potassium chloride (KCl)	Sigma-Aldrich	P5405, CAS:7447-40-7
Magnesium sulfate heptahydrate (MgSO_4_.7H_2_O)	HiMedia	GRM683, CAS:10034-99-8
Glucose	Sigma-Aldrich	G7021, CAS:50-99-7
Pancreatin	Sigma-Aldrich	P1750; CAS: 8049-47-6
Collagenase A	Roche	10103586001; CAS: 9001-12-1
Gelatine from bovine skin	Sigma-Aldrich	G9391; CAS: 9000-70-8
Tetramethylrhodamine ethyl ester perchlorate	Sigma-Aldrich	87917; CAS:115532-52-0
DAPI (4′,6-diamidino-2-phenylindole dihydrochloride)	Sigma-Aldrich	D9542; CAS:28718-90-3
5-bromo-2′-deoxyuridine	Sigma-Aldrich	B5002, CAS: RN 59-14-3

**Deposited data**		

Data obtained with this protocol	Bongiovanni et al.^1^	N/A

**Experimental models: Organisms/strains**		

Postnatal day 0–1 mice: strain C57BL/6N	Charles River Laboratories	Strain C57BL/6N

**Other**		

Cylindrical magnetic stirring bars 12 mm × 4.5 mm	Biosigma	046033
50 mL self-standing tube	Biosigma	CL477

## Materials and equipment

**Table:** Cardiac cell adhesion medium

Reagent	Final concentration	Amount
DMEM F12 with stable Glutamine and 15 mM HEPES	81%	40.5 mL
Fetal Bovine Serum (FBS)	10%	5 mL
Horse Serum (HS)	5%	2.5 mL
Penicillin/Streptomycin (100x)	1%	0.5 mL
Glutamine (100x)	1%	0.5 mL
MEM Non-essential Amino acids (100x)	1%	0.5 mL
Sodium Pyruvate (100 mM)	1%	0.5 mL
**Total**	**–**	**50 mL**

Store at 4°C for up to 2 weeks.

**Table:** Cardiomyocyte culture medium

Reagent	Final concentration	Amount
DMEM F12 with stable Glutamine and 15 mM HEPES	91%	45.5 mL
Horse Serum (HS)	5%	2.5 mL
Penicillin/Streptomycin (100x)	1%	0.5 mL
Glutamine (100x)	1%	0.5 mL
MEM Non-essential Amino acids (100x)	1%	0.5 mL
Sodium Pyruvate (100 mM)	1%	0.5 mL
**Total**	**–**	**50 mL**

Store at 4°C for up to 2 weeks.

**Table:** ADS Buffer (pH 7.4)

Reagent	Final concentration	Amount
NaCl	106 mM	3.095 g
HEPES	20 mM	2.385 g
Na_2_HPO_4_	0.8 mM	0.057 g
KCl	5.3 mM	0.197 g
MgSO_4_.7H_2_O	0.4 mM	0.049 g
Glucose	5 mM	0.451 g
H_2_O	**–**	up to 500 mL

Store at 4°C for up to 2 weeks. See “preparation of ADS buffer” above for details.

**Table:** Pancreatin and collagenase enzyme solution

Reagent	Final concentration	Amount
Collagenase A	0.45 mg/mL	22.5 mg
Pancreatin	1 mg/mL	50 mg
Cold ADS buffer		50 mL

Use fresh and keep in ice. See “preparation of enzymatic digestion solution (collagenase-pancreatin enzyme solution)” above for details.

**CRITICAL:** Collagenase (CAS: 9001-12-1) has the following hazard statements: H315 (skin irritation), H319 (eye irritation), H334 (allergy or asthma symptoms or breathing difficulties if inhaled), H335 (respiratory tract irritation). Take the following precautions when handling this reagent: avoid breathing dust during preparation, use wear gloves protection and face mask, wash skin thoroughly after handling. Pancreatin (CAS: 8049-47-6) has the following hazard statements: H315 (skin irritation), H317 (skin sensitization), H319 (eye irritation), H334 (allergy or asthma symptoms or breathing difficulties if inhaled); H335 (respiratory tract irritation). Take the following precautions when handling this reagent: avoid breathing dust during preparation, use wear gloves protection and face mask, wash skin thoroughly after handling.

### Alternative reagents

This protocol outlines the specific reagents used in our published studies.^1^^,^^8^ However, there is flexibility in selecting certain components, such as powders for preparing the ADS buffer and reagents used for cardiomyocyte medium (e.g., serums, antibiotics, sodium pyruvate, ...). Below are some tested alternatives.

• Fetal bovine serum (Aurogene AU-S1810).
• Horse serum (Aurogene AU-S0900).
• Penicillin-streptomycin solution (Aurogene AU-L0022).
• Glutamine (Aurogene AU-L0022).
• Non-essential amino acids (Aurogene AU-X0557).
• Sodium pyruvate (Aurogene AU-L0642).

We strongly recommend using DMEM-F12 medium supplemented with 15 mM HEPES.

The choice of the 50-mL self-standing tubes is flexible, but ensure they have a conical inner bottom to facilitate the magnetic bar to tilt during rotation, which improves the gentle movement of the hearts.

The type of agitator is also flexible; however, it should have a flat bottom, be suitable for use in a 37°C incubator, and be capable of operating at speeds below 100 RPM.

## Step-by-step method details

### Setting the equipment

**Timing: 1.5 h**

This step outlines the setup of the equipment required for the isolation of cardiac muscle cells. The procedure described is for a single isolation involving a maximum of 8–10 hearts. For processing more hearts, perform parallel isolations.

1. Prepare Enzyme Solution:
  a. Prepare 50 mL of fresh, sterile Collagenase A/Pancreatin enzyme solution in cold ADS buffer and keep on ice.
  b. Aliquot enzyme solution into four tubes (8 mL each) and store on ice.
  c. Heat each tube to 37°C for approximately 10 min before use to activate the enzymes.
2. Prepare Magnetic Stirring Bar:
  a. Wash a cylindrical magnetic stirring bar (12 × 4.5 mm) with absolute ethanol, then allow it to dry under a biological hood.
  b. Transfer the magnetic bar into a 50 mL self-standing tube with a conical inner bottom.
3. Prepare cardiac cell adhesion medium and warm it to 37°C.
4. Prepare Digestion Tubes:
  a. Set up four 15 mL tubes for the subsequent digestion steps.
  b. Warm heat-inactivated horse serum and pipette 1 mL into each tube. Horse serum is used to inactivate enzymes after each digestion step.
5. Place a magnetic stirrer on a shelf in an incubator at 37°C and 5% CO_2_.
6. Cover the wells of cell culture plates with cold 0.1% gelatin solution (100 μL for 96-well plates and 1 mL for 12-well plates) and warm them in an incubator at 37°C and 5% CO_2_.

***Note:*** Warming the gelatin-coated plates for at least 1 h (Step 6) is necessary for optimal cardiomyocyte seeding. This can be done concurrently with the isolation procedure.

**CRITICAL:** For optimal adhesion of cardiomyocytes, ensure that the gelatin coating is done on polystyrene cell culture plates.

### Cardiac cell isolation and seeding

**Timing: 3 h**

The isolation procedure involves four sequential enzymatic and mechanical digestion steps in an incubator set to 37°C and 5% CO_2_, facilitating the disaggregation of the cardiac tissue and isolation of cardiac cells.

7. Heart extraction:
  a. Sacrifice neonatal (postnatal day-0 or 1) mice by decapitation and extract the hearts.
  b. Transfer the hearts into a Petri dish containing cold ADS buffer (pH 7.4).
  c. Cut the hearts at the ventricular level to facilitate washing and subsequent digestion.
  d. Remove excess blood by gently pressing the hearts with curved-pointed tweezers.

**CRITICAL:** Sterilize the scissors and tweezers with 70% ethanol before use. Dry them thoroughly to prevent tissue exposure to ethanol. Complete this step quickly to minimize exposure to a non-sterile environment. The next step should be performed under a biological laminar flow hood (see troubleshooting 1).

8. First digestion step (washing and pre-digestion phase):
  a. Transfer the hearts to the inside edge of a 50 mL self-standing tube containing the cylindrical magnetic bar.
  b. Add 8 mL of pre-warmed Collagenase A and Pancreatin enzyme solution, ensuring the hearts are at the bottom of the tube.
  c. Place the tube on a magnetic stirrer in an incubator set to 37°C and 5% CO_2_, ensuring slow agitation for 10 min (Methods video S1).
  d. After 10 min, slowly remove and discard 7 mL of solution using a serological pipette.

***Note:*** The first digestion step serves as a washing and pre-digestion phase to remove blood and debris before enzymatic digestion. Discarding this first digestion ensures subsequent enzymatic treatments are more effective in isolating specific cell types, such as cardiomyocytes, with higher yield and viability.

**CRITICAL:** Ensure the magnetic bar spins at a slow speed (Methods video S1). Excessive agitation can accelerate tissue digestion, potentially compromising cardiomyocyte integrity (see troubleshooting 2). Maintain a gentle up-and-down motion of the hearts.

**CRITICAL:** Ensure that the hearts move freely above and below the magnetic bar and do not stick to the bottom or top of the tube. Adjust the agitation speed if necessary.

**CRITICAL:** Set the pipettor to the minimum aspiration speed and leave 1 mL of enzyme solution at the bottom of the tube to avoid aspirating heart tissue.

9. Enzymatic and mechanical digestion:
  a. Add 7 mL of pre-warmed Collagenase A and Pancreatin enzyme solution to the 50 mL self-standing tube.
  b. Place the tube back in the incubator at 37°C and 5% CO_2_ on the magnetic stirrer, ensuring gentle agitation for 20 min (Methods video S1).
  c. After digestion, carefully remove the supernatant using a serological pipette and transfer it to a 15 mL tube containing 1 mL of horse serum (to inactivate the enzymes).
  d. Centrifuge the tube at 400 *g* (RCF) with brake 6 for 5 min.
  e. Discard the supernatant by inverting the tube.
  f. Resuspend the cardiac cell pellet in 2 mL of adhesion medium and place it in the cell culture incubator set to 37°C and 5% CO_2_ with the tube cap slightly loosened to allow gas exchange.
  g. Perform a total of 4 digestions of 20 min each and collect 4 tubes containing isolated cardiac cells by repeating the last 6 sub-steps (a-f) described above (see troubleshooting 3).

***Note:*** For 3 to 5 hearts, use a reduced volume of enzyme solution (4 mL).

**CRITICAL:** The procedure is less efficient with fewer than 3 hearts.

**CRITICAL:** For each digestion, set the pipettor to the minimum aspiration speed and leave 1 mL of enzyme solution at the bottom of the tube to avoid aspirating heart tissue when transferring the digested supernatant.

10. Cardiac cell pooling and resuspension:
  a. Pool the isolated cardiac cells from the fourth digestion tubes into a single 15 mL tube.
  b. Centrifuge at 400 *g* with brake 6 for 5 min and discard the supernatant.
  c. Resuspend the cell pellet in 4 mL of adhesion medium.

***Note:*** For isolation from fewer than 5 hearts, resuspend in 2 mL of adhesion medium.

**CRITICAL:** Avoid vigorous or prolonged resuspension of the cardiac cells at all steps to preserve cell integrity.

11. Cardiac cell counting and seeding:
  a. Count the isolated cardiac cells and seed them in 0.1% gelatin-coated cell culture plates with cardiac cell adhesion medium.

***Note:*** For immunofluorescence analysis seed 35,000 cells per well in 96-well plates. For protein and gene expression analysis, seed 400,000 to 500,000 cells per well in 12-well plates (see troubleshooting 4).

**Figure 1:**
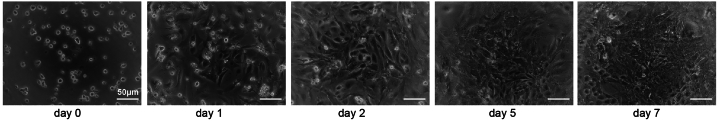
Neonatal cardiac cell maintenance after isolation Neonatal cardiac cells seeded at a density of 35,000 cells per well in a 96-well plate. Images were captured immediately after seeding (day 0) and 1-, 2-, 5- and 7-days post seeding; scale bars, 50 μm. This figure is related to the section “expected outcomes.”

12. Cardiomyocyte enrichment (optional):

The above-described procedure enables the isolation of a mixed population of cardiac cells, including cardiomyocytes and stromal cells (Figure 1). To enrich for cardiomyocytes before seeding, use the immuno-magnetic cell sorting MACS Neonatal Isolation System (Miltenyi Biotec #130-100-825), following the manufacturer’s protocol (https://static.miltenyibiotec.com/asset/150655405641/document_0dt75odmel4vj4f978opc7ca4k?content-disposition=inline) and as described in our study.^1^ Alternatively, pre-plating can be employed as a strategy to selectively adhere non-cardiomyocyte cells, allowing for the enrichment of cardiomyocytes in the supernatant for subsequent seeding.

Methods video S1. Neonatal cardiac tissue digestion, related to steps 8 and 9 Hearts from postnatal day 0–1 mice were enzymatically and mechanically disaggregated using a magnetic bar and stirrer within a 37°C and 5% CO_2_ incubator.

### Cardiomyocyte maintenance

**Timing: 2 days**

This section outlines the adhesion timing and culture conditions required for freshly isolated neonatal cardiac cells.

13. Allow primary isolated cardiac cells to adhere for 2 days after seeding in the adhesion medium at 37°C and 5% CO_2_.

***Note:*** After 2 days, cardiomyocytes should appear well-adhered and actively beating (Figure 1 and Methods video S2).

14. Replace the cardiac cell adhesion medium with a cardiomyocyte culture medium (FBS-free). At this stage, cardiomyocytes retain proliferative potential (Methods video S3), and the cardiac cell culture can be treated with substances of interest or exposed to specific environmental conditions.

***Note:*** The FBS-free medium is not selective for cardiomyocyte growth, but the absence of FBS limits the excessive proliferation of stromal cells. Cardiomyocytes can be distinguished in culture by their characteristic contractions under microscopic observation (Methods video S2).

***Note:*** If treatment duration exceeds 2 days, it is advisable to replace the cardiomyocyte culture medium with fresh medium.

**CRITICAL:** Avoid exposing the cells to rapid or significant temperature changes during the adhesion and treatment phases (see troubleshooting 5).

Methods video S2. Beating cultured neonatal cardiomyocytes, related to step 13 Two days after seeding, murine neonatal cardiomyocytes were recorded to document their beating activity. The video was taken at 20X magnification.

Methods video S3. Proliferation of cultured neonatal cardiomyocytes, related to step 14 Neonatal cardiomyocytes were cultured *in vitro*, allowed to adhere for two days, stained with a mitochondrial fluorescent dye (TMRE) and analyzed time-lapse imaging for 16 h. The circle points at a cardiomyocyte undergoing cell division; scale bars, 50 μm.

## Expected outcomes

**Figure 2:**
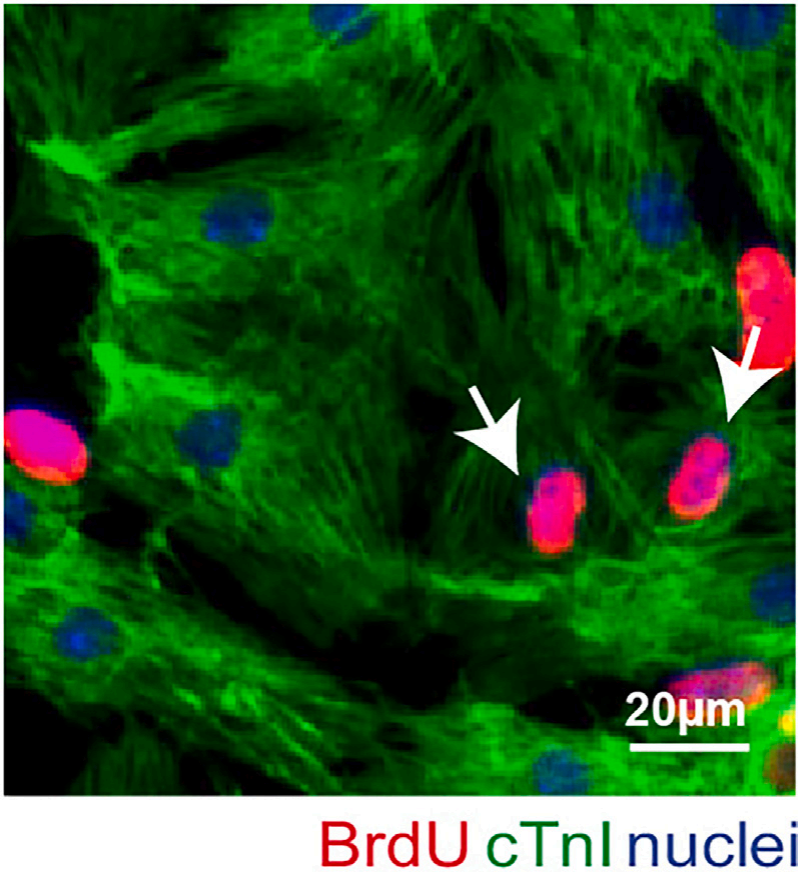
Proliferative ability of isolated neonatal cardiomyocytes Neonatal cardiomyocytes were cultured *in vitro*, allowed to adhere for two days, and analyzed for BrdU incorporation (red) on the second day post-adhesion. Cardiomyocytes were stained for cardiac Troponin I (cTnI) (green). A representative image is provided; arrows point at proliferating cardiomyocytes; scale bars, 20 μm. This figure is related to the section “expected outcomes.”

This procedure, optimized for mouse models, typically yields approximately 1 × 10^6^ cardiac cells per neonatal mouse heart. After the adhesion phase, the isolated viable cardiomyocytes are characterized by their strong adhesion to the culture surface and typically appear elongated and flattened. During this period, most cardiomyocytes exhibit beating activity (Methods video S2) and some retain proliferative potential, as evidenced by duplication events in time-lapse videos (Methods video S3)^1^^,^^8^ proliferative assays (approximately 3%–8% of cardiomyocytes are proliferating in a 48-h BrdU assay) (Figure 2). Cultured cardiomyocytes remain viable for up to one week post-isolation (Figure 1). Beyond this time, the culture becomes suboptimal due to the overgrowth of stromal cells and, unfortunately, most cardiomyocytes do not survive the trypsinization and replating procedures.

## Limitations

A limitation of the procedure is the challenge in obtaining a pure cardiomyocyte culture without the use of a specific enrichment kit or additional purification techniques.

The efficiency of cardiomyocyte isolation may be impacted by several factors, including manual handling during resuspension steps (e.g., overly vigorous), suboptimal conditions (e.g., excessive agitation of the magnetic stirrer), or prolonged exposure to cold temperatures. These factors can reduce cell yield and decrease the number of viable, beating cardiomyocytes.

It is also worth noting that while polystyrene cell culture plates facilitate cardiomyocyte adhesion, they may introduce higher background signals in immunofluorescence assays compared to glass cell culture plates, which are not optimal for cardiomyocyte adhesion.

## Troubleshooting

### Problem 1

Contamination of isolated cells.

### Potential solution

The initial steps of the procedure, such as heart extraction and washing, are conducted in non-sterile conditions, which can increase the risk of contamination. To minimize this risk, ensure all instruments (e.g., forceps and tweezers) are sterilized with 70% ethanol before use. Use a sterile Petri dish and ADS buffer for cardiac tissue washing. For the subsequent isolation steps, work under aseptic conditions in a biosafety cabinet. Ensure sterility by using freshly filtered reagents and ethanol-cleaned instruments throughout the entire process.

### Problem 2

Improper rotation speed of the magnetic bar (related to steps 8 and 9).

### Potential solution

The rotation speed of the magnetic stirrer may need adjustment depending on the specific equipment and the number of hearts being processed. Excessive agitation can accelerate tissue digestion, however, compromising cardiomyocyte integrity and reducing cell yield. Conversely, insufficient rotation may slow the digestion process, also resulting in lower cell yields. To optimize the process, set the magnetic stirrer to the lowest speed that allows the magnetic bar to rotate smoothly and facilitate the up-and-down motion of the hearts (Methods video S1). This ensures proper tissue digestion while maintaining cardiomyocyte viability.

### Problem 3

The cardiac tissue remains undigested following four digestion steps (related to step 9).

### Potential solution

In some instances, not all the heart tissue may be fully digested, particularly when isolating cells from a higher number of hearts (e.g., 8 to 10). If only small pieces of tissue remain, attempt to gently resuspend them with a serological pipette before proceeding to sub-step c of step 9 to ensure complete disaggregation.

If larger pieces of heart are still present, consider continuing with a fifth digestion cycle.

### Problem 4

**Figure 3:**
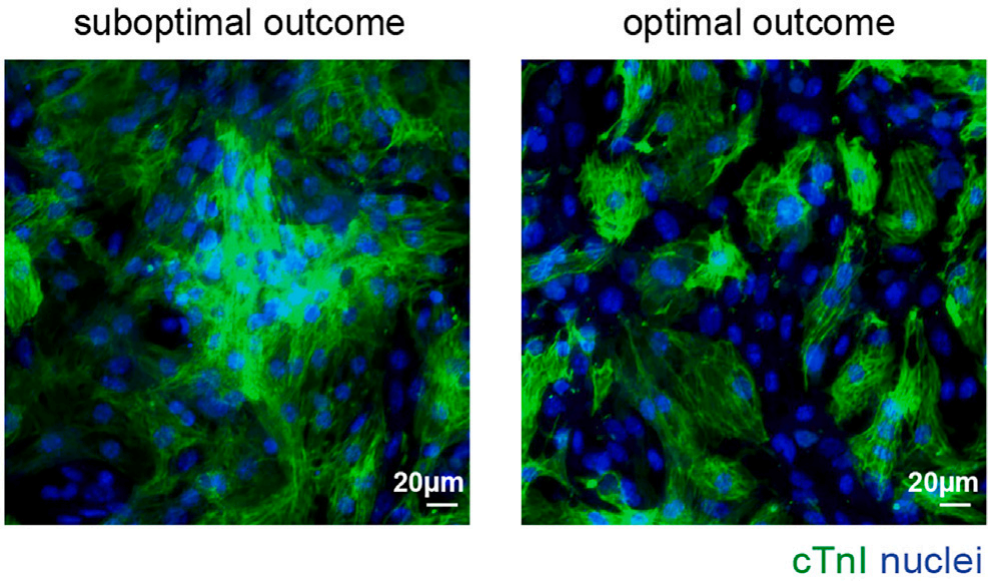
Immunofluorescence analysis of cardiomyocytes isolated from neonatal mice Neonatal cardiomyocytes were cultured *in vitro*, allowed to adhere for two days, and analyzed by immunofluorescence for cardiac Troponin I (cTnI) (green) on the second day post-adhesion. Representative images display an example of both optimal and suboptimal cell seeding outcomes; scale bars, 20 μm. This figure is related to the section “troubleshooting 4.”

Cardiomyocytes cluster in one area of the well, leading to focal beats (related to step 11) (see Figure 3).

### Potential solution

Cardiomyocytes, being relatively large cells, tend to settle and cluster in the center of the well after seeding, especially in 96-well plates. This can lead to uneven cell distribution, making analysis more challenging, particularly for assays like immunofluorescence where markers (e.g., proliferation markers) need to be assessed evenly across the well. To address this, immediately after seeding, gently shake the plate and tap its edges to promote even distribution of the cells within the wells (Figure 1). Alternatively, after seeding, place the plates on a slowly shaking platform in a 37°C, 5% CO_2_ incubator to ensure more uniform spreading of the cells across the well surface.

### Problem 5

Interruption of cardiomyocyte beating as consequence of prolonged exposure to cold temperatures (related to step 14).

### Potential solution

Cardiomyocytes are highly sensitive to temperature fluctuations. Exposure to cold temperatures, even briefly, during the adhesion or treatment phases can result in a slowing or temporary interruption of their beating. Strategies for avoiding rapid temperature changes include:

• Maintain stable conditions: keep seeded cells in an incubator at 37°C and 5% CO_2_ at all times, particularly during the critical two-day adhesion period.
• Pre-warm solutions and equipment: ensure that any solutions and equipment used are pre-warmed to the appropriate temperature to avoid sudden temperature changes.
• Handle with care: use temperature-controlled transport systems when moving seeded plates to prevent disruptions in cardiomyocyte activity. If cardiomyocytes experience a slowdown in beating due to temperature fluctuations, they generally resume normal activity once returned to the incubator and allowed to re-acclimate.

## Resource availability

### Lead contact

Further information and requests for resources and reagents should be directed to Gabriele D’Uva and will be fulfilled by the lead contact, Gabriele D’Uva (gabrielematteo.duva2@unibo.it).

### Technical contact

Technical questions on executing this protocol should be directed to Chiara Bongiovanni and will be answered by the technical contact, Chiara Bongiovanni (chiara.bongiovanni2@unibo.it).

### Materials availability

This protocol did not generate new unique reagents.

### Data and code availability

This protocol did not generate new datasets.

## Acknowledgments

This work has received funding from the European Union - NextGenerationEU through the Italian Ministry of University and Research under PNRR - M4C2-I1.3 Project PE_00000019 ‘‘HEAL ITALIA’’ to G.D. (CUP J33C22002920006) and was supported by the 10.13039/501100003196Italian Ministry of Health (RC-2024-2790614). The views and opinions expressed are those of the authors only and do not necessarily reflect those of the European Union or the European Commission. Neither the European Union nor the European Commission can be responsible for them.

## Author contributions

C.B., C.M., and G.D. wrote and edited the manuscript; C.B., F.S., S.D.P., I.D.B., S.B., and C.M. validated the protocol; C.B. generated the figures and videos; G.D. supervised the work.

## Declaration of interests

The authors declare no competing interests.
